# FastQ Screen: A tool for multi-genome mapping and quality control

**DOI:** 10.12688/f1000research.15931.2

**Published:** 2018-09-17

**Authors:** Steven W. Wingett, Simon Andrews

**Affiliations:** 1 Bioinformatics, Babraham Institute, Cambridge, CB22 3AT, UK

**Keywords:** Bioinformatics Contamination FastQC Illumina Metagenomics NGS QC Sequencing

## Abstract

DNA sequencing analysis typically involves mapping reads to just one reference genome. Mapping against multiple genomes is necessary, however, when the genome of origin requires confirmation. Mapping against multiple genomes is also advisable for detecting contamination or for identifying sample swaps which, if left undetected, may lead to incorrect experimental conclusions. Consequently, we present FastQ Screen, a tool to validate the origin of DNA samples by quantifying the proportion of reads that map to a panel of reference genomes. FastQ Screen is intended to be used routinely as a quality control measure and for analysing samples in which the origin of the DNA is uncertain or has multiple sources.

## Introduction

In general, reaching sound conclusions from sequencing experiments requires the origin of a sample to be identified correctly prior to mapping. To reduce the risk of contaminants leading to incorrect inferences, it is advisable to map sequencing results against not only the expected reference genome but also against reasonable sources of contamination. Common reasons for contamination include amplifying the wrong target molecule, unwanted DNA being present in reagents used in library generation, carry-over from samples previously loaded onto a sequencing machine or sample swaps.

**Figure 1.**
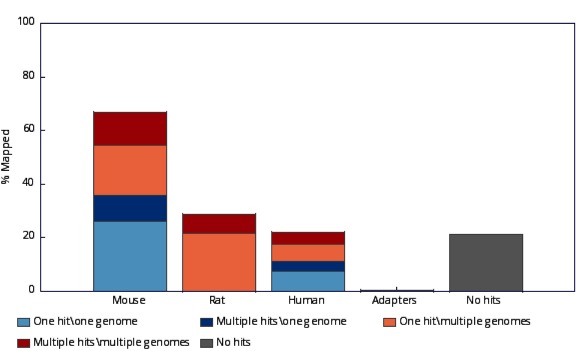
Graphical output from FastQ Screen after mapping a publicly available RNA-Seq sample (SRR5100711) against several reference genomes. Reads either i) mapped uniquely to one genome only (light blue), ii) multi-mapped to one genome only (dark blue), iii) mapped uniquely to a given genome and mapped to at least one other genome (light red), or iv) multi-mapped to a given genome and mapped to at least one other genome (dark red). The reads represented by blue shading are significant since these are sequences that align only to one genome, and consequently, if are observed in an unexpected genome they suggest contamination.

The tool utilises either Bowtie ^1^, Bowtie 2 ^2^ or BWA ^3^, as preferred by the user, to map reads against pre-specified genomes. FastQ Screen presents the mapping results in both text and graphical formats, thereby allowing the user to confirm the genomic origin of a sample or identify sources of DNA contamination. The tool summarises the proportion of reads that map to a single genome or to multiple genomes. In addition, it reports whether those alignments are to a unique position, or to more than one location, within the genome of interest ( Figure 1).

FastQ Screen functionality is generally independent of the laboratory protocol followed and so can be used to analyse genomic DNA, RNA-Seq ^4^, ChIP-Seq or Hi-C experiments. In addition, FastQ Screen is compatible with Bismark ^5^, and so can also be used to process bisulfite sequence data.

Other tools exist with similar functionality to FastQ Screen, most notably Multi Genome Alignment (MGA) ^6^. FastQ Screen has a number of advantages over these tools, including directly reporting the proportion of multi-mapping reads, thereby helping identify DNA populations rich in low-complexity sequences. Another benefit of our program is the capability to create filtered FASTQ files. FastQ Screen is also the only quality control (QC) tool that aligns reads to multiple bisulfite reference genomes.

## Methods

### Implementation

The program utilises a short read sequence aligner to map FASTQ reads against pre-defined reference genomes. The tool records against which genome or genomes each read maps and summarises the results in graphical and text formats.

### Operation

We coded FastQ Screen in Perl and made use of the CPAN module GD::Graph for the generation of summary bar plots. The software requires a functional version of Bowtie, Bowtie 2 or BWA, and should be run on a Linux-based operating system. FASTQ Screen uses Plotly to enable visualisation of results in a web browser. The tool takes as input a text configuration file and FASTQ files, which are sub-sampled by default to 100,000 reads to reduce running times, and then mapped to a panel of pre-specified genomes.

## Use cases

**Preliminary sequencing QC:** FastQ Screen provides preliminary evidence on whether a sequencing run has been successful, as demonstrated in Figure 1, which shows results using a publicly available RNA-Seq sample ( SRR5100711) labelled as mouse. The software processed the deposited FASTQ file to generate summary results in text, HTML and PNG format. As expected, the dataset contained a substantial proportion of reads that mapped only to the mouse genome, and although a sizeable proportion of reads mapped to both the mouse and rat genomes, that may have also been expected considering the close evolutionary relationship between those two species. Of concern, however, was the discovery that 11.4% of the reads mapped solely to the human genome, suggesting the sample was contaminated. This may prove problematic if human-derived reads that also align to the mouse reference genome are not removed, since differences between mouse samples may then actually reflect the variation in the degree of contamination between the samples rather than genuine biological differences. Very few reads aligned to adapter sequences which was an encouraging observation.

**Identifying sample origin from a range of alternatives:** FastQ Screen was recently used by researchers to identify the origin of the clothes of the Tyrolean Iceman (popularly named Ötzi), a famous 5,300 year old natural mummy discovered in 1991 in the Italian Ötztal Alps. By screening sequences against probable sources of preserved leathers, the research team showed that the iceman’s hat came from Brown Bear, his quiver from Roe deer and his loincloth came from sheep ^7^. In a similar fashion, FastQ Screen has been used to determine the animal origin of vellum found in 13th century Bibles ^8^.

**Filtering results:** FastQ Screen can also be used to filter reads mapping (or not mapping) to specified genomes. This has numerous applications, most typically to remove DNA contaminants, as exemplified by a recent clinical microbial metagenomics study in which nucleic acids were extracted from porcine faeces ^9^. FastQ Screen was then used to filter-out host sequences, and the remaining reads were then mapped, leading to the identification of over 1,600 bacterial and Archaea species and strains of virus.

In contrast, in some experiments the source of contamination may be completely unpredictable and so we have incorporated a setting in which all unsuccessfully mapped reads are written to a FASTQ format output file. This may then be used by other resources, such as BLAST, to determine the origin of those sequences.

## Summary

Since its release, FastQ Screen has been used to analyse a myriad of sequencing datasets. We initially envisioned the software as a QC tool to complement our related program FastQC, but we subsequently used the software to confirm the origin of samples and added functionality for filtering FASTQ reads. The program may be used in conjunction with several common aligners, including Bismark for processing bisulfite libraries. FastQ Screen has been incorporated by other groups into bioinformatics workflows, was reimplemented in the recently released QC tool Aozan ^10^, and is compatible with MultiQC ^11^, a tool to aid comparison of samples with respect to a large number of QC metrics.

## Software availability

FastQ Screen is available from: https://www.bioinformatics.babraham.ac.uk/projects/fastq_screen

Source code available from: https://github.com/StevenWingett/FastQ-Screen

Archived source code as at time of publication: https://doi.org/10.5281/zenodo.1346672 ^12^

License: GNU GPL 3.0
