# Supplementary material for: FastQ Screen: A tool for multi-genome mapping and quality control
Source: F1000Res. 2018 Sep 17;7:1338. Originally published 2018 Aug 24. [Version 2] doi: 10.12688/f1000research.15931.2 (PMC6124377; doi:10.12688/f1000research.15931.2)
Supplement: Peer review history

# Peer review: Referee response for version 2

**DOI:** 10.5256/f1000research.17815.r38403

**Published:** 2018-09-18

**Authors:** Stéphane Le Crom, Laurent Jourdren

**Affiliations:** 1 Sorbonne Université, Univ Antilles, Univ Nice Sophia Antipolis, Paris, France; 2 Sorbonne Université, UMS Omique, Plateforme Post-génomique de la Pitié-Salpêtrière, Paris, France; 3 Institut de biologie de l’Ecole normale supérieure (IBENS), Ecole normale supérieure, Paris, France

With this second version the authors answer all the concerns we raised about their FastQ Screen article. We will approve it for indexing.

We have read this submission. We believe that we have an appropriate level of expertise to confirm that it is of an acceptable scientific standard.

# Peer review: Referee response for version 1

**DOI:** 10.5256/f1000research.17398.r37623

**Published:** 2018-09-17

**Authors:** Matthew D. Teasdale

**Affiliations:** 1 BioArCh, University of York, York, UK

In this paper Wingett and Andrews describe FastQ Screen a program for quality control and source species identification. The paper is well written with clear example use cases and I am very happy to recommend FastQ Screen for indexing. 

 I have personally used FastQ Screen for over 6 years and now consider it to be an essential part of my analysis pipelines. The documentation for the program is excellent and it is under active development.

I have read this submission. I believe that I have an appropriate level of expertise to confirm that it is of an acceptable scientific standard.

# Response

**Published:** 2018-09-17

**Authors:** Steven Wingett

**Affiliations:** Babraham Institute, UK

We are delighted that FastQ Screen has proven useful in your research and we hope it will remain part of your analysis pipeline as we add new features to the software.

# Peer review: Referee response for version 1

**DOI:** 10.5256/f1000research.17398.r37622

**Published:** 2018-09-06

**Authors:** Stéphane Le Crom, Laurent Jourdren

**Affiliations:** 1 Sorbonne Université, Univ Antilles, Univ Nice Sophia Antipolis, Paris, France; 2 Sorbonne Université, UMS Omique, Plateforme Post-génomique de la Pitié-Salpêtrière, Paris, France; 3 Institut de biologie de l’Ecole normale supérieure (IBENS), Ecole normale supérieure, Paris, France

When dealing with multiple high throughput sequencing experiments, especially for core facilities, you need to pay great attention to quality controls. Contaminations from different species you are working with are one of the potential problems you can encounter. The FastQ Screen software as been designed by Steven Wingett and Simon Andrews in order to solve this drawback. Using different mapping softwares, FastQ Screen allows to identify from the reads present in your samples the different species they came from. The graphical and text outputs provided, detailed information on the potential level of contamination obtained.

 The software objectives are clearly explained just as the way to use it and how to interpret its outputs. FastQ Screen source code is available through GitHub and a documentation is provided on the authors’ website.

 This software is publicly available since several years and is used today by many genomics laboratories. The tool is very stable, the command line help is easy to understand and we not found any issue when we launch it in all our tests. Finally, the output report is informative and very clear.

 FastQ Screen is a must have tool for everyone working with multiple species samples or who want to prevent unpredicted contamination of its samples.

 Remarks

 1. In order to more clearly explain the way FastQ Screen is working, more information should be provided on pre-defined reference genomes. How can it be chosen before running FastQ Screen. Is there a limitation among the number of genomes selected? Is there a significant impact on software running time according to the number or size of reference genomes selected? Is there a list of already available pre-defined reference genomes? Does it works using subset sequence database or with the whole genome?

 2. It could also be interesting to get some information about the running time of several analyses. What are the specifications required for the computer needed to run FastQ Screen? I didn’t findd this information through the documentation.

 3. It seems that FastQ Screen when processing large dataset (FASTQ files more than 100 million reads) use a large amount of memory as it store the identifier of each read. It may be useful to advise users in documentation about this issue.

 Minor remarks

 1. For future evolution of FastQ Screen software it could be interesting to provide a clue for the “No Hits” reads obtained. In the paper the authors suggest to run BLAST analyses. Perhaps there is a way to use a subset of “NoHits” reads in order to guess where the possible contamination is coming from?

 2. The link to the “Babraham Bioinformatics download page” in the documentation “https://www.bioinformatics.babraham.ac.uk/projects/fastq_screen/_build/html/index.html#download” is not working

We have read this submission. We believe that we have an appropriate level of expertise to confirm that it is of an acceptable scientific standard, however we have significant reservations, as outlined above.

# Response

**Published:** 2018-09-17

**Authors:** Steven Wingett

**Affiliations:** Babraham Institute, UK

Thank you for your detailed feedback. Both you and the reviewer Dr Hamilton pointed out that FastQ Screen would be better served if we made pre-made genome indices available.  As described in our response to Dr Hamilton, FastQ Screen now has a --get_genomes option to obtain Bowtie2 genome indices.  We have also provided more information in the documentation ( https://www.bioinformatics.babraham.ac.uk/projects/fastq_screen/_build/html/index.html), explaining how a user may create the desired aligner index files.

We now also mention in the documentation that FastQ Screen has an upper limit to the number of genomes that may be used.  This value, which is 32, is the result of how the script records read/genome mapping data as a 32-bit variable.  This limit far exceeds every task we have performed with FastQ Screen.  Should more genomes be required at a future date however, we would be able to modify the code to increase the maximum allowed value.

The time taken to process a dataset varies substantially depending on the input data and specified parameters.  For example, larger files take longer to process than those with fewer reads.  Similarly, screening against more genomes will increase processing times, as will the complexity and size of the genomes to which the aligner maps FASTQ reads.  Using FastQ Screen in the default quality control (QC) mode, in which only a subset of the data is processed, is significantly quicker than using the tool to filter a dataset.  Screening bisulfite libraries is also a more computationally intensive task and therefore takes significantly longer to complete.  In contrast, times may be reduced by muti-threading submitted jobs.  The hardware of a system will of course also substantially impact running times, as will the competition for system resources from the jobs being run concurrently with FastQ Screen.

There are similar points to consider when evaluating memory overheads.   Most notably, running the program in QC mode will require substantially less memory than filtering a dataset.  The software needs to hold in memory whether a read maps to any of the reference genomes.  In QC mode, this will only be necessary for approximately 100,000 reads, but when filtering this will be required for every read, simultaneously, in the FASTQ file – and FASTQ files may comprise hundreds of millions of reads.

To help the user make sense of these considerations, we have now included in the documentation a report of the memory requirements and time taken to process different data files, using different parameters.  Obviously, it is impossible to cover every scenario, but as a general rule using the tool to QC a dataset should take minutes whereas filtering a large dataset may take several hours.

We added extra information in the documentation pertaining to the system requirements necessary to run FastQ Screen.  To summarise these, FastQ Screen should be run on an up-to-date Linux operating system that has Perl installed and has a working version of either Bowtie, Bowtie2 or BWA installed.

So far as we can tell, the following web link is functional: https://www.bioinformatics.babraham.ac.uk/projects/fastq_screen/_build/html/index.html#download.  In case there is any confusion, this is an anchor to the “Download” section in the documentation and not a link to the software download page.

Thank you for bringing to our attention the need for follow-on support for reads that map to no genomes (extracted when using the --no_hits parameter).  We intend to address this in future FastQ Screen releases by adding new functionality to the software.

# Peer review: Referee response for version 1

**DOI:** 10.5256/f1000research.17398.r37624

**Published:** 2018-09-04

**Authors:** Ian J. Donaldson

**Affiliations:** 1 Faculty of Biology, Medicine and Health, University of Manchester, Manchester, UK

FastQ Screen by Wingett and Andrews is a tool to map a sample of sequenced reads against a panel of reference genomes. 

 The tool is comprehensively documented and is available from the authors' web site and via Github.    

 I have personally used this tool since 2011 and it is incorporated in the quality control pipeline for our core facility on all sequencing runs.  The tool has been used to detect contamination from a panel of commonly used genomes, and to estimate the contribution of sequence from mixed genome samples. It is also used to detect rRNA contamination in RNA-seq protocols. Recently the ability of FastQ Screen to filter individual reads has been incorporated into our single cell analysis pipeline.  

 The article is clear, well explained, and gives interesting use cases.

Minor correction:

 In the legend of Figure1 -

 'Reads either i) mappped' should be 'Reads are either i) mapped'

 'ii) mapped uniquely' should be 'iii) mapped uniquely'

 '(light red) or multi-mapped' should be '(light red), or iv) multi-mapped'

I have read this submission. I believe that I have an appropriate level of expertise to confirm that it is of an acceptable scientific standard.

# Response

**Published:** 2018-09-17

**Authors:** Steven Wingett

**Affiliations:** Babraham Institute, UK

Thank you for your comments and we are pleased that you find FastQ Screen useful in your research.  We have updated the manuscript to correct the typographical errors.

# Peer review: Referee response for version 1

**DOI:** 10.5256/f1000research.17398.r37620

**Published:** 2018-08-29

**Authors:** Russell S. Hamilton

**Affiliations:** 1 University of Cambridge, Cambridge, UK

Wingett and Andrews present FastQ Screen for mapping sequencing reads to multiple genomes with the goal of identifying the genome of origin. FastQ Screen is well documented, open source and freely available via GitHub and their own website.

 I have been using the software routinely as part of my NGS pipelines for several years and find it to be an invaluable QC  tool. Sample mix ups, if from different species, are easily detected. I have also found FastQ Screen useful as a proxy indicator of rRNA contamination in total RNA-Seq, due to rRNAs being similar between related species, they show up as multi-genome aligned reads, thus identifying samples for further processing.

 I therefore have no reservations in recommending FastQ Screen for indexing.

**Suggestions:**

1. Each lab or facility will have their own unique requirements for genomes to screen against, but having a suggested “starter set” of genomes may ease the burden of installation / configuration for first time users. The bowtie website has some pre-made common, bowtie indexed, genomes for convenient download ( http://bowtie-bio.sourceforge.net/bowtie2/index.shtml). Or more comprehensively, Illumina’s iGenome project contains a wide range of bowtie/bowtie2/bwa indexed genomes ( http://support.illumina.com/sequencing/sequencing_software/igenome.ilmn).
2. One of the most useful features of FastQ Screen is its compatibility with MultiQC, where multiple samples are plotted together for assessment of entire sequencing runs or batches. Mentioning this in the documentation would alert users to this very useful feature.

I have read this submission. I believe that I have an appropriate level of expertise to confirm that it is of an acceptable scientific standard.

# Response

**Published:** 2018-09-17

**Authors:** Steven Wingett

**Affiliations:** Babraham Institute, UK

We agree with the excellent suggestion to create pre-built genomes for users.  Indeed, the latest version of FastQ Screen (v0.13.0) now has a new option (--get_genomes) which instructs the script to download commonly used pre-built Bowtie2 reference genomes deposited on the Babraham Bioinformatics website.  Along with the reference genomes, FastQ Screen also downloads a configuration file, which it subsequently edits to list the full path of the downloaded genomes as stored on the user’s machine.  This setup should be ready-to-use and the selection of genomes should suit most requirements.

 We have updated the documentation to alert users that our tool is compatible with MultiQC.
